# A pretraining domain decomposition method using artificial neural networks to solve elliptic PDE boundary value problems

**DOI:** 10.1038/s41598-022-18315-4

**Published:** 2022-08-17

**Authors:** Jeong-Kweon Seo

**Affiliations:** Institute of Data Science, Korea University, 145 Anam-ro, Seongbuk-gu, Seoul, 02841 South Korea

**Keywords:** Engineering, Mathematics and computing, Physics

## Abstract

Developing methods of domain decomposition (DDM) has been widely studied in the field of numerical computation to estimate solutions of partial differential equations (PDEs). Several case studies have also reported that it is feasible to use the domain decomposition approach for the application of artificial neural networks (ANNs) to solve PDEs. In this study, we devised a pretraining scheme called smoothing with a basis reconstruction process on the structure of ANNs and then implemented the classic concept of DDM. The pretraining process that is engaged at the beginning of the training epochs can make the approximation basis become well-posed on the domain so that the quality of the estimated solution is enhanced. We report that such a well-organized pretraining scheme may affect any NN-based PDE solvers as we can speed up the approximation, improve the solution’s smoothness, and so on. Numerical experiments were performed to verify the effectiveness of the proposed DDM method on ANN for estimating solutions of PDEs. Results revealed that this method could be used as a tool for tasks in general machine learning.

## Introduction

Partial differential equations (PDEs) contain principled physical laws and govern the object’s physical or engineering systems. For instance, PDEs are mathematically analyzed to understand scientific and physical phenomena such as heat, electricity, fluid dynamics, and so on. However, due to the analytic complexity of a given PDE, it is usually impossible to have their explicit solutions. In correspondence, in the science of numerical solutions of PDEs, many studies have been conducted to efficiently solve problems by applying various discretization methods such as the finite difference method^1^, finite element method^2^, and other high-order methods such as the spectral method^3^. Eventually, as the era of the artificial intelligence approaches^4^ with their applications, for example, to cognitive science^5^, biomedical science^6,7^, ecology^8,9^, manufacturing^10^, and more, many numerical methodologies of computational physics based on machine learning techniques have also been actively studied, including radial basis function networks^11^, Gaussian Kernels^12^, least squares support vector machine^13^, dimension reduction methods^14–16^, deep learning with Monte Carlo method^17^, autoencoder^18^, and convolutional neural networks^19^.

On the other hand, Li et al.^20^ have briefly summarized the use of machine learning into three categories: (1) implementing physics into machine learning by generating a dataset of physical laws^21^, (2) designing a physics-guided machine learning method with physics-guided model as a black box^22^, and (3) imposing physics into the training process, such as the method of “physics-informed neural networks (PINN)”^23^. Among these various types of machine learning technologies, this paper will focus on artificial neural networks (ANN) of the third category^24–26^. Unlike most of other state-of-the-art machine learning techniques that lack robustness or fail to guarantee convergence with small data regimes, the method of PINN encodes physical information into the training process and well generalizes to the right solution even when only a few training examples are available^23^.

Regarding the development of ANN-based PDE solvers, Lagaris et al.^27,28^ have made early contributions to the application of ANN towards solving systems of PDEs. Using analogues of their model, subsequent research has been performed on empirical methodologies to use ANNs for the calculus of PDEs, including irregular boundary problems, arbitrary boundary problems^29,30^, initial-boundary value problems^31^, time-constrained optimal control problems^32^, the methods of unsupervised learning^33^, immersed boundary problem^34^, and a constrained backpropagation approach^35^.

In 2020, devising a domain decomposition methodology (DDM), Jagtap et al.^36^ proposed conservative physics-informed neural networks to solve Burgers’ equations, incompressible Navier–Stokes equations, and compressible Euler equations. Among many variations of ANN models, in Refs.^36,37^, the authors implemented PINN-based domain decomposition methodologies and showed their approximations’ effectiveness by *hp*-refinement. They divided their computational domain into several subdomains and constructed the main loss function by employing weight parameters on terms of boundary/initial conditions, the residual from governing equations and interfaces, and applied their method to forward and inverse problems of several challenging nonlinear PDEs. Since *hp*-refinement technologies have been one of the main concerns for research performing numerical analysis of PDEs^38–40^, their breakthroughs employing the ANN-based decomposition of the domain could not be regarded as anything other than impressive.

Solving PDE by ANN models, many studies have developed efficient technologies as listed in above. However, there is no model that employs a concept of pretraining technique. As the main contribution of our proposed method in our work, we proposed a pretraining scheme to improve the performance of a given model of ANN to solve elliptic PDEs.

In this study, as another approach for an ANN-based DDM, we developed a pretraining scheme DDM. The pretraining process that is engaged in at the beginning of the training epochs helps the approximation basis get well-posed on the domain so that the accuracy of the estimated solution is enhanced, i.e., the speed of approximation is improved rather than non-pretrained schemes. We report that such a well-organized pretraining scheme is effective as an NN-based PDE solver. In our work, we did not focus on handling disadvantages of existing methods. Instead, we focused on developing an ad-hoc technology by proposing a pretraining scheme so that it could be applied to any given existing ANN models to enhance their numerical performances.

The proposed pretraining scheme consists of two parts. The first part is to train along with governing equations without any engagement of the boundary or subdomain’s interfacial conditions. The second part is to train for neighboring subdomains to be flattened, i.e., a subdomain’s function of the estimation is compelled to be zero at data points located on neighboring subdomains. In this article, we call the first part the *smoothing* process and the second part the *basis reconstruction* (BR) process. With numerical experiments, we show that as the proposed pretraining process continues, the estimated solution’s output of the fine-tuning improves. Moreover, we report that the BR process helps the numerical basis of the ANN become well-posed to approximate sorts of high degrees of polynomials that are in solutions as the BR process makes the basis of ANN of a subdomain closely gather on its region such that the initial state of the ANN, at the starting point of the fine-tuning, has high smoothness.

Other than existing methodologies of the NN-based domain decomposition method that employ hyperparameters within the loss function, our proposed method consists of a scheme of pretraining and fine-tuning approach that allows us to use the scheme in various architectures of deep-learning algorithms. As the main contribution of our proposed method, the pretraining process that is engaged at the beginning of the training epochs helps the approximation basis being well-posed on the domain so that the quality of the estimated solution is enhanced. We report that such well-organized pretraining scheme may give effectiveness to any NN-based PDE solver as we get a speed-up in approximation and improvement in the solution’s smoothness and so on.

In “Methods” section, we introduce our domain decomposition method for ANNs. In “Numerical experiments” section, we present numerical results of using our method to solve elliptic boundary value PDEs and the XOR problem as a demonstration of the method’s performance for application to the general machine learning task. We then discuss properties of our proposed method. In “Conclusion” section, we present some conclusions drawn from our study.

## Methods

### The feedforward multi-layered neural networks and the system of PDEs

**Figure 1:**
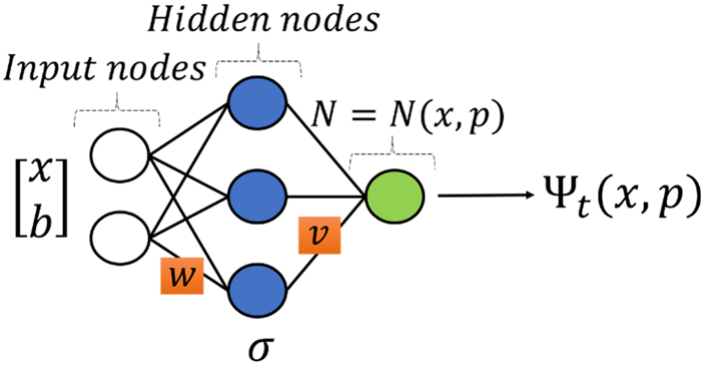
$d$-Dimensional ANN structure.

Let the architecture of the multi-layered ANN (abbreviated as ANN thereafter) be given as follows:

1 $$N\left(x,p\right)={v}^{T}\sigma \left(w\overline{x }\right),$$

where $v\in {\mathbb{R}}^{H}$ and $w\in {\mathbb{R}}^{H\times \left(d+1\right)}$ denote learnable weights, $p$ represents them as general parameters, the input variable is denoted by $\overline{x }$, an extension of $x$ such that $\overline{x }={\left(x,b\right)}^{T}$ with a constant $b$ for the computation of the bias weight in $w$, and “$\sigma :{\mathbb{R}}^{H}\to {\mathbb{R}}^{H}$” is a component-wise activation function. In our study, we employed the sigmoidal activation function. The $d$-dimensional problem structured by $N\left(x,p\right)$ is illustrated in Fig. 1.

Following^28^, we considered the following system of PDEs:

2 $$G\left(x,\Psi \left(\text{x}\right),\nabla\Psi \left(x\right),{\nabla }^{2}\Psi \left(x\right)\right)=0, x \in\Omega ,$$

where $\Psi \left(x\right)$, with $x=\left(x(1),\cdots ,x(d)\right)\in {\mathbb{R}}^{d}$, was the solution of Eq. (2) on a domain $\Omega \subset {\mathbb{R}}^{d}$ which satisfied essential boundary conditions specified on the boundary $\partial\Omega$ of $\Omega$. In our numerical examples, we supposed $\Omega$ to be an open convex polygon in ${\mathbb{R}}^{2}$ such that $\overline{\Omega }$ was the union of a finite number of polyhedra.

To satisfy Eq. (2) and approximate $\Psi \left(x\right)$, let us define a trial solution ${\Psi }_{t}\left(x\right):$

3 $${\Psi }_{\text{t}}\left(x,p\right)=A\left(x\right)+B\left(x\right)N\left(x,p\right),$$

where $A\left(x\right)$ satisfies the boundary condition such that $\Psi \left(x\right)=A\left(x\right), \forall x\in \partial\Omega$, and $N\left(x,p\right)$ is the single-hidden-layered ANN. The scalar function $B\left(x\right)$ is constructed to satisfy $B\left(x\right)N\left(x,p\right)=0, \forall x\in {\partial\Omega }_{D}$, where ${\partial\Omega }_{D}$ is the portion of $\partial\Omega$ where the essential boundary condition is imposed. For details of techniques on how to impose constraints, please refer to Refs.^28,29^.

To estimate the trial solution ${\Psi }_{t}\left(x,p\right)$ solving the system of Eq. (2), we defined discretization $\widehat{\Omega }$ of $\overline{\Omega }$ such that $\widehat{\Omega }=\left\{{\xi }_{j}\in\Omega ;j=1,\dots ,m\right\}$. Then Eq. (2) is in the form of the collocation method^3^ as follows:

4 $$\underset{p}{\text{min}}\frac{1}{2}\sum \limits_{j=1}^{m}{\left(G\left({\xi }_{j},{\Psi }_{\text{t}}\left({\xi }_{j},p\right),\nabla {\Psi }_{\text{t}}\left({\xi }_{j},p\right),{\nabla }^{2}{\Psi }_{\text{t}}\left({\xi }_{j},p\right)\right)\right)}^{2},\, {\xi }_{j} \in \widehat{\Omega }.$$

To train weight parameters of the minimization problem in Eq. (4), we applied the backpropagation method used in gradient descent^27,28,35^.

### Implementation of the gradients and training

To compute the gradient of ${\Psi }_{\text{t}}\left(\xi ,p\right)$, $\xi \in \widehat{\Omega }$, we discretized the 1st- and 2nd-order derivatives by the backward and midpoint Euler scheme, respectively, such that

5 $$\left\{\begin{array}{l}\frac{{\partial\Psi }_{\text{t}}\left(\xi ,p\right)}{\partial x(i)}=\frac{{\Psi }_{\text{t}}\left(\xi ,p\right)-{\Psi }_{\text{t}}\left({\xi }^{i,-\delta } ,p\right)}{\delta }\\ \frac{{{\partial }^{2}\Psi }_{\text{t}}\left(\xi ,p\right)}{\partial x{\left(i\right)}^{2}}=\frac{{\Psi }_{\text{t}}\left({\xi }^{i,+\delta },p\right)-2{\Psi }_{\text{t}}\left(\xi ,p\right)+{\Psi }_{\text{t}}\left({\xi }^{i,-\delta },p\right)}{{\delta }^{2}}\end{array}\right.,$$

where for a positive real number $\delta >0$, ${\xi }_{i,-\delta }$ and ${\xi }_{i,+\delta }$ were defined as varying the $i$th component of $\xi$ by $\pm \delta$, i.e., ${\xi }^{i,-\delta }=(\xi (1),\xi (2),\dots ,\xi (i)-\delta ,\dots ,\xi (d))$, ${\xi }^{i,+\delta }=(\xi \left(1\right),\xi \left(2\right),\dots ,\xi \left(i\right)+\delta ,\dots ,\xi (d))$. Note that, for the implementation of numerical experiments in this paper, we set $\delta ={10}^{-6}$. Using the numerical approximation theory of derivatives, one may set it as sufficiently small.

As for our example problem of Eq. (2), the Poisson equation is given as follows:

6 $$\left\{ {\begin{array}{*{20}l} { - \nu \nabla^{2} \Psi \left( x \right) - F\left( x \right) = 0,} \hfill & {x \in {\Omega }} \hfill \\ {\Psi \left( x \right) = 0,} \hfill & {x \in \partial {\Omega }} \hfill \\ \end{array} } \right.,$$

where $F\left(x\right)$ is the source function.

To estimate the solution of Eq. (6), we discretized the minimization problem of Eq. (6) as follows:

7 $$\underset{p}{\text{min}}\frac{1}{2}\sum \limits_{j=1}^{m}{\left(-\nu \sum \limits_{i=1}^{d}\frac{{{\partial }^{2}\Psi }_{\text{t}}\left({\xi }_{j},p\right)}{\partial x{\left(i\right)}^{2}}-F({\xi }_{j})\right)}^{2}, {\xi }_{j} \in \widehat{\Omega }.$$

Now, by employing the gradient descent method with a suitable learning rate, we obtained the iterative solution of Eq. (7). For weight parameters $w$ and $v$ of $p$, using Eqs. (5) and (7), their $n$ th epoch’s updates of gradients $\Delta {w}^{(n)}$ and $\Delta {v}^{(n)}$ are given by the chain rule of the derivatives of Eq. (7) for ${w}^{(n)}$ and ${v}^{(n)}$, respectively, as follows:

8 $$\begin{aligned} {\Delta }w_{k,l}^{\left( n \right)} = & - \frac{\nu }{{\delta^{2} }}\mathop \sum \limits_{i = 1}^{d} { }\left( { - \nu \mathop \sum \limits_{i = 1}^{d} \frac{{\partial^{2} {\Psi }_{{\text{t}}} \left( {\xi_{j} ,p^{\left( n \right)} } \right)}}{{\partial x\left( i \right)^{2} }} - F\left( {\xi_{j} } \right)} \right) \\ & \times \left( {B\left( {\xi_{j}^{i, + \delta } } \right)v_{k}^{\left( n \right)} \sigma_{k}^{^{\prime}} \left( {w^{\left( n \right)} \overline{{\xi_{j}^{i, + \delta } }} } \right)\overline{{\xi_{j}^{i, + \delta } }} \left( l \right) - 2B\left( {\xi_{j} } \right)v_{k}^{\left( n \right)} \sigma_{k}^{^{\prime}} \left( {w^{\left( n \right)} \overline{{\xi_{j} }} } \right)\overline{{\xi_{j} }} \left( l \right) + B\left( {\xi_{j}^{i, - \delta } } \right)v_{k}^{\left( n \right)} \sigma_{k}^{^{\prime}} \left( {w^{\left( n \right)} \overline{{\xi_{j}^{i, - \delta } }} } \right)\overline{{\xi_{j}^{i, - \delta } }} \left( l \right)} \right), \\ {\Delta }v_{k}^{\left( n \right)} = & - \frac{\nu }{{\delta^{2} }}\mathop \sum \limits_{i = 1}^{d} { }\left( { - \nu \mathop \sum \limits_{i = 1}^{d} \frac{{\partial^{2} {\Psi }_{{\text{t}}} \left( {\xi_{j} ,p^{\left( n \right)} } \right)}}{{\partial x\left( i \right)^{2} }} - F\left( {\xi_{j} } \right)} \right) \\ & \times \left( {B\left( {\xi_{j}^{i, + \delta } } \right)\sigma_{k} \left( {w^{\left( n \right)} \overline{{\xi_{j}^{i, + \delta } }} } \right) - 2B\left( {\xi_{j} } \right)\sigma_{k} \left( {w\overline{{\xi_{j} }} } \right) + B\left( {\xi_{j}^{i, - \delta } } \right)\sigma_{k} \left( {w^{\left( n \right)} \overline{{\xi_{j}^{i, - \delta } }} } \right)} \right), \\ \end{aligned}$$

where ${\sigma }_{k}^{^{\prime}}$ is the derivative of the $k$ th component of $\sigma$.

### The pretraining domain decomposition method for ANNs

In our study, the given computational domain was divided into several non-overlapping smaller computational regions over which the governing PDEs were inherited. We call these small-sized computational domains as subdomains of our domain decomposition method.

Now, the core of the DDM scheme we proposed began with the flux-continuity on the interfaces of the subdomains. After that, the construction of the basis related to the solutions on the subdomains was considered. To illustrate the scheme, we defined a pair of consecutive neighborhood subdomains $\widehat{{\Omega }_{r}}$, $\widehat{{\Omega }_{s}}\subset \widehat{\Omega }$ where $\widehat{{\Omega }_{r}}$ and $\widehat{{\Omega }_{s}}$ were disjoint except for their interfaces. Trial functions ${\Psi }_{\text{t},\text{r}}\left(\cdot ,{p}_{r}\right)$ and ${\Psi }_{\text{t},\text{s}}\left(\cdot ,{p}_{s}\right)$ were given for $\widehat{{\Omega }_{r}}$ and $\widehat{{\Omega }_{s}}$, respectively.

#### Flux-continuity on interfaces

The training for the interfacial flux-continuity was divided into two parts: the nodal continuity and the continuity of the normal directional derivatives.

1. Nodal continuity:
  9 $${\Psi }_{\text{t},\text{r}}\left(\zeta ,{p}_{r}\right)-{\Psi }_{\text{t},\text{s}}\left(\zeta ,{p}_{s}\right)=0, \zeta \in \widehat{{\Omega }_{r}}\cap \widehat{{\Omega }_{s}.}$$
2. Continuity of the outward normal directional derivatives:
  10 $$\frac{\partial {\Psi }_{\text{t},\text{r}}\left(\zeta ,{p}_{r}\right)}{\partial \overrightarrow{{n}_{r}}}+\frac{\partial {\Psi }_{\text{t},\text{s}}\left(\zeta ,{p}_{s}\right)}{\partial \overrightarrow{{n}_{s}}} =0, \zeta \in \widehat{{\Omega }_{r}}\cap \widehat{{\Omega }_{s.}}$$

For normal directional derivatives in Eq. (10), we applied direct differentiation on ${\Psi }_{\text{t},\text{r}}\left(\zeta ,{p}_{r}\right)$ and ${\Psi }_{\text{t},\text{s}}\left(\zeta ,{p}_{s}\right)$ for respect to normal directions on their domains. Neglecting the subscript of $r$ or $s$, we have

11 $$\frac{\partial {\Psi }_{\text{t}}\left(\zeta ,p\right)}{\partial x\left(l\right)}=\frac{\partial A\left(\zeta \right)}{\partial x\left(l\right)}+\frac{\partial B\left(\zeta \right)}{\partial x\left(l\right)}{v}^{T}\sigma \left(w\overline{\zeta }\right)+B(x)\sum \limits_{k=1}^{{N}_{h}}{v}_{k}^{T}{w}_{k,l}{\sigma }^{^{\prime}}\left(w\overline{\zeta }\right),$$

Hence,

12 $$\frac{\partial {\Psi }_{\text{t}}\left(\zeta ,p\right)}{\partial \overrightarrow{n}}=\nabla {\Psi }_{\text{t}}\left(\zeta ,p\right)\cdot \overrightarrow{n},$$

where $\overrightarrow{n}$ is the outward directional normal vector on the interface.

#### Construction of the basis on subdomains

**Figure 2:**
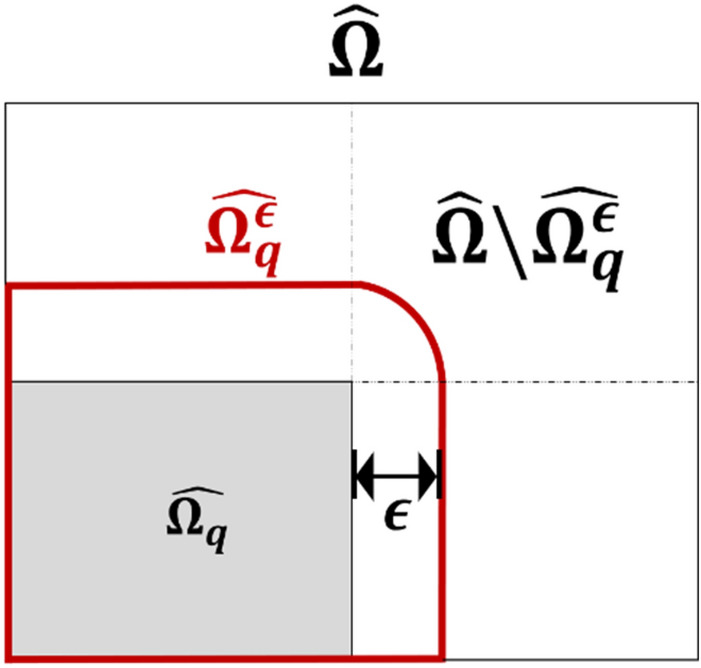
A 2D rectangular domain $\widehat{\Omega }$ and $\widehat{{\Omega }_{q}^{\upepsilon }}$.

As the key subject of our study, a pre-processing approach was taken for the model of multi-subdomains by proposing a pretraining scheme that could initialize training weight parameters well. We aimed to enhance the convergence power. This pretraining can be divided into two parts:

1. *Pretraining for the loss function on each subdomain (smoothing process)* In this step, we strengthened the convergence power interior of each subdomain except for terms of the flux-continuity. From this, we performed an early estimation of the smoothness interior before the interfacial flux-continuity exchanges. The pretraining scheme implemented to optimize the problem is given as follows:
  13 $$\underset{{p}_{q}}{\text{min}}\frac{1}{2}\sum \limits_{j=1}^{{m}_{q}}{\left(-\nu \sum \limits_{i=1}^{d}\frac{{{\partial }^{2}\Psi }_{\text{t}}\left({\xi }_{j},{p}_{q}\right)}{\partial x{\left(i\right)}^{2}}-F({\xi }_{j})\right)}^{2}, {\xi }_{j} \in \widehat{{\Omega }_{q}} ,$$
  where the subscript $q$ denotes the arbitrary index of subdomains.
2. *Pretraining to impel the basis into each subdomain (basis reconstruction (BR) process)* In this step, we aim to train the network to nullify the basis arising in the neighboring subdomains. We hypothesize that if the function-shaping basis exists outside of the interface, it may weaken the power of the polynomial basis that shapes the interior estimations. Hence, to prevent the basis from being non-zero outside of the interfaces, we performed nullification pretraining such that
  14 $${\Psi }_{\text{t},\text{q}}\left(\eta ,{p}_{q}\right)=0, \eta \in \widehat{\Omega }\backslash \widehat{{\Omega }_{q}^{\upepsilon }},$$
  where the $\widehat{{\Omega }_{q}^{\upepsilon }}$ was the $\upepsilon$-expansion of $\widehat{{\Omega }_{q}}$ for a given positive number $\epsilon >0\in {\mathbb{R}}$ defined by
  15 $$\widehat{{\Omega }_{q}^{\upepsilon }}=\left\{\eta \in \widehat{\Omega }\backslash \widehat{{\Omega }_{q}}: \left|\eta -\xi \right|\ge \epsilon \text{for all} \xi \in \widehat{{\Omega }_{q}}\right\}.$$
  The definition of $\widehat{{\Omega }_{q}^{\upepsilon }}$ is illustrated in Fig. 2. Assuming that a two-dimensional (2D) rectangular domain $\widehat{\Omega }$ is given, the gray-shaded region depicts $\widehat{{\Omega }_{q}}$ and the region enclosed by a red curve represents $\widehat{{\Omega }_{q}^{\upepsilon }}$.

Note that the above two parts of the pretraining occurred subsequently in each epoch. The main purpose of the pretraining scheme is to enhance computational smoothness of basis functions made of activation functions. As we nullified solutions outside of a given subdomain, we assumed that the smooth region in the basis function moved near the region of the subdomain so that the well-posed basis into the domain might prevent leakage of the computational basis’ smoothness off the domain.

**Figure 3:**
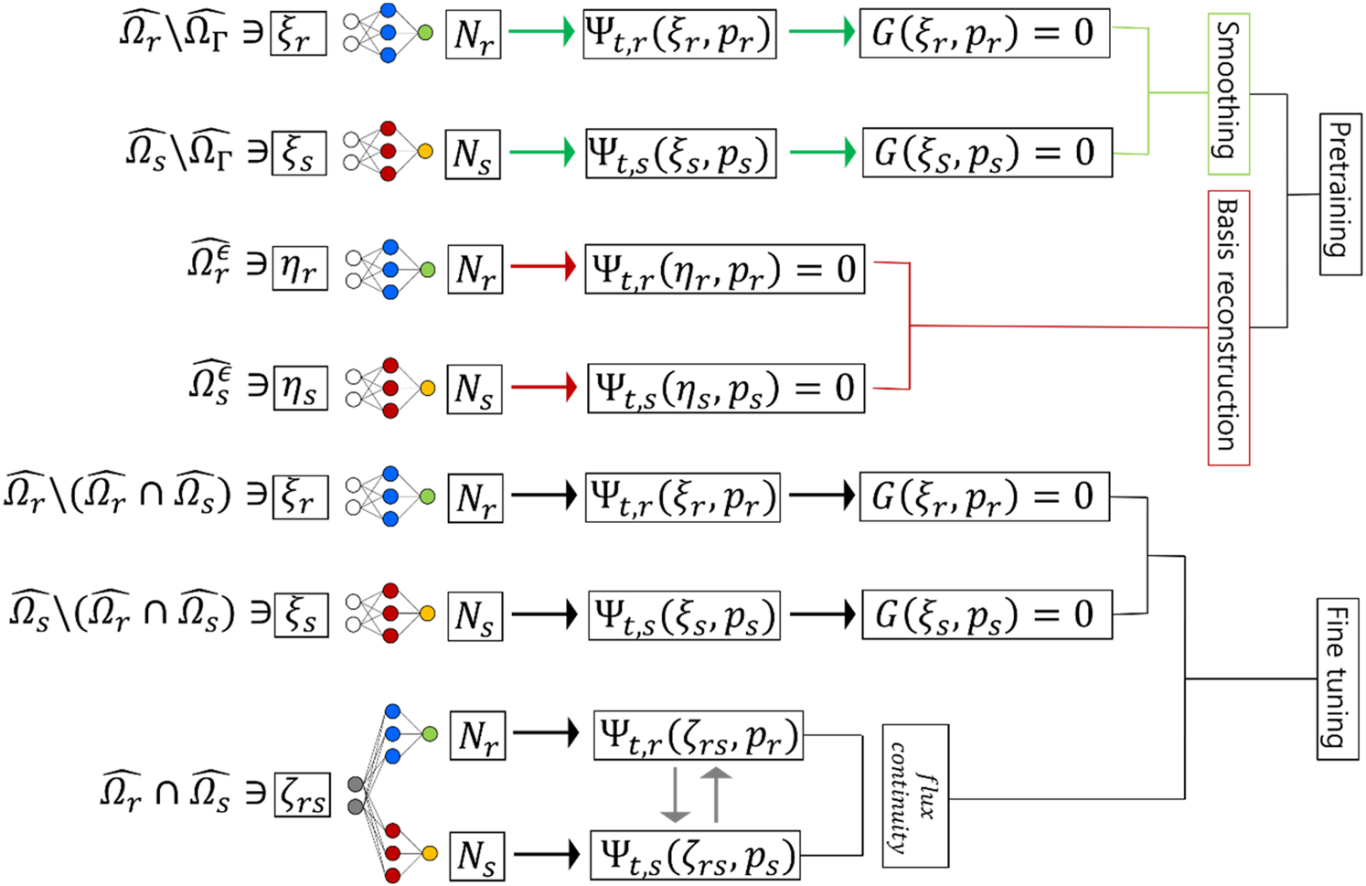
The structure of the pretraining version of ANN-based DDM.

The proposed ANN version of DDM is illustrated in Fig. 3.

## Numerical experiments

The first numerical experiment we performed was a preliminary study to investigate the pretraining’s effect on one-dimensional (1D) problems. We applied the smoothing process as pretraining without basis reconstruction or nullification for the Poisson problem with the Dirichlet boundary condition.

Note that in our work, the benchmark algorithm, which is conceptually the same as the one with the algorithm introduced in Ref.^36^, i.e., a DDM model without pretraining process, was one of the recently proposed algorithms to apply the traditional DDM method calculated by preserving the flux continuity between subdomains to ANN as it is (refer to refs^36,37^.).

### 1D problem: effect of smoothing

The 1D Poisson equation with Dirichlet boundary condition is:

16 $$\left\{\begin{array}{l}-\nu \Delta u=f \,in\, \Omega \\ u=0 \,on\, \partial \Omega \end{array},\right.$$

where the solution $u$ satisfies equations over the domain $\Omega =\left[\text{0,2}\right]$ and the boundary $\partial\Omega$ of $\Omega$ is $\partial\Omega =\Omega \backslash (\text{0,2})$. As a test solution, we let $u=u\left(x\right)$, for $x\in\Omega$, such as

17 $$u\left(x\right)={e}^{{x}^{2}}\text{sin}\left(2\pi x\right).$$

**Figure 4:**
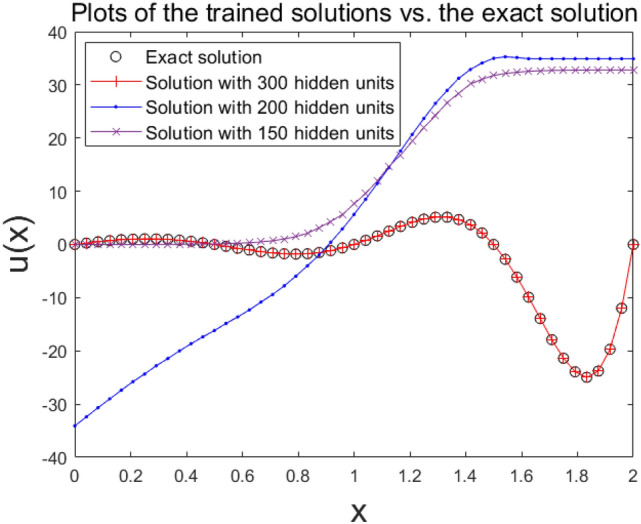
Solutions with 150, 200, and 300 hidden units from the 1D domain with $3\times {2}^{4}$ nodes as estimated by the single domain method.

Figure 4 shows plots of the estimated solution made from the ordinary single domain method. They were compared to the exact solution. To compare, we verified the use of hidden units which we set to be 150, 200, and 300. To organize the set of training data, we discretized the domain equi-spatially into $3\times {2}^{4}$ nodes. Among estimations, only the case of using 300 hidden units gave a good approximation to the solution. Note that when updating neural weights in the training, we conducted the smallest batch model that updated weights for each input data, like the stochastic batch mode.

**Figure 5:**
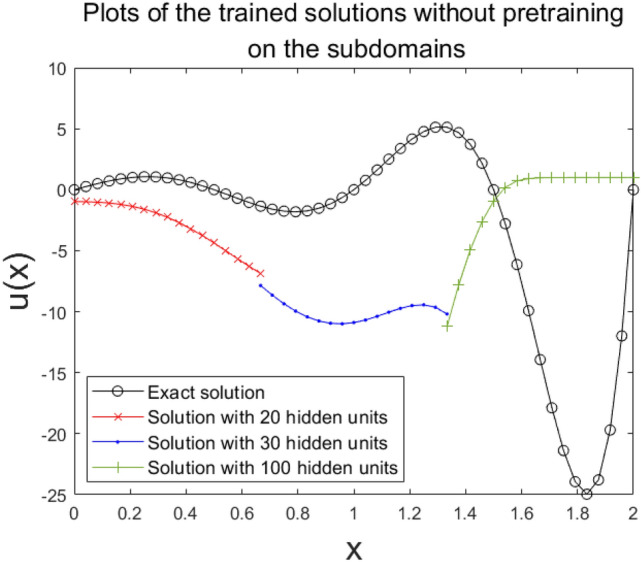
The solution without pretraining process on the 1D domain is decomposed into three subdomains with ${2}^{4}$ nodes in each subdomain, where the total number of hidden units is 150.

Now, to assess the computing power effectiveness of the DDM model, we conducted training with a three-subdomain method without any pretraining process. Figure 5 shows a plot of these results. Here, hidden units are set to be 20, 30, and 100 on each subdomain. Hence, the total number of the hidden units is 150. As show in Fig. 5, preset hidden units of the DDM failed to estimate the solution. Thus, the ordinary scheme of the DDM with ANN did not improve the results further in this case.

**Figure 6:**
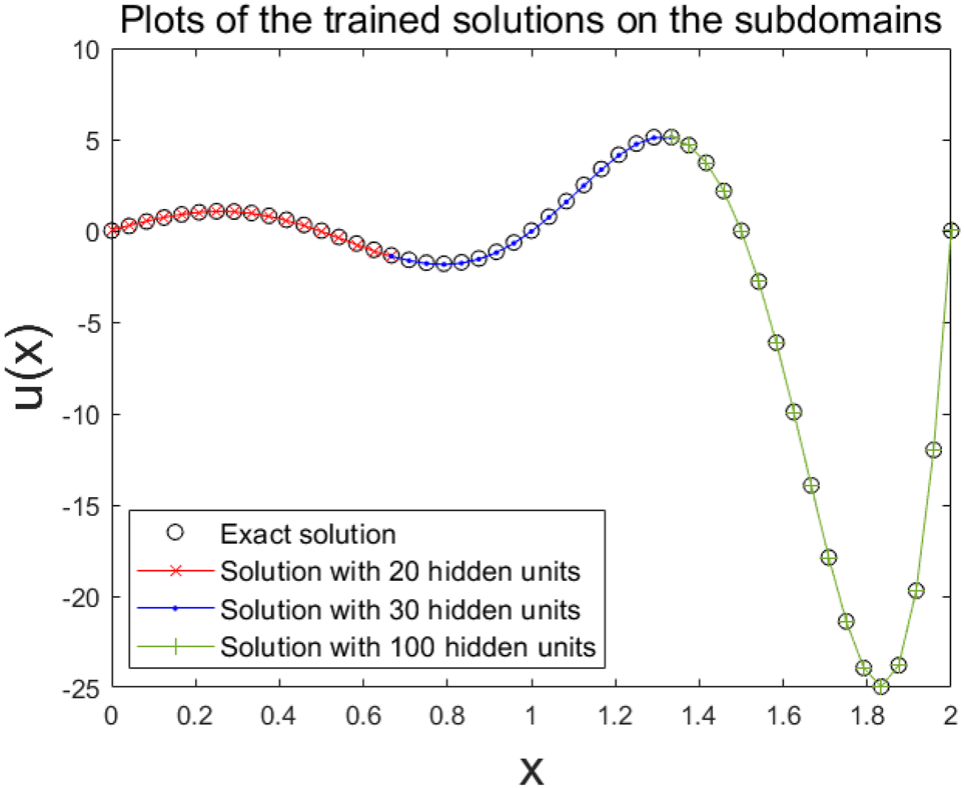
The solution with the smoothing pretraining process on the 1D domain is decomposed into three subdomains with ${2}^{4}$ nodes for each subdomain, where the total number of hidden units is 150.

**Figure 7:**
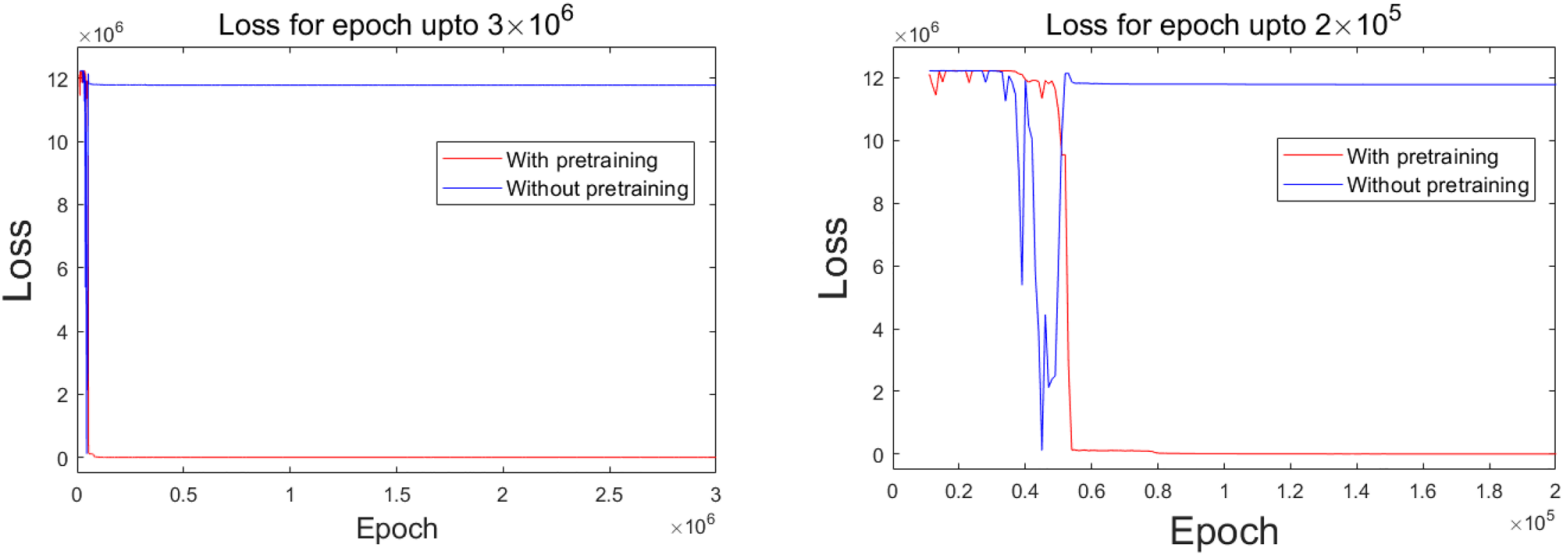
A comparison of the decay of the cost function’s loss for the training of the solution on the 1D domain decomposed into three subdomains with ${2}^{4}$ nodes for each subdomain, where the total number of hidden units is 150.

Next, to improve DDMs by using smoothing for pretraining, we applied it to the same structure of subdomains with the same sets of hidden units. The pretraining used 10,000 epochs. For fine-tuning, the ratio of the training epochs at the boundary, subdomain’s interface, and the governing equation was set to be 20:2:1, i.e., the training took place at boundary nodes for 20 epochs, then for 2 epochs at the interface, and then 1 epoch for the governing equation on interior nodes of each subdomain. Here, the number of training epochs at the boundary was set relatively high since we noted that the structure of our ANN was not equipped with an ansatz employed to hold the boundary condition. We propose that further training at the boundary will allow estimations to be stable and maintain their analytic uniqueness. The resulting configuration of the estimated solution and the plots of the progression of the loss versus epoch are shown in Figs. 6 and 7, respectively. It was revealed that the model of smoothing well approximated the exact solution in Eq. (17). However, the single domain method and the ordinary approach of the DDM failed to make an accurate estimate (Figs. 6, 7). This demonstrates that such highly-oscillatory solutions in Eq. (17), which the ordinary single domain method hardly solves with lower amounts of hidden units, are more effectively solved by the DDM model with its effective alignment of the subdomains’ numerical basis delivered from the DDM’s inherent domain separation scheme property.

Note that, for our numerical results, the learning rate is set to be ${10}^{-6}$. From our preliminary tests, the convergence rate deteriorated for some scales of the learning rates much greater than ${10}^{-6}$. Even for DDM solutions, in the subregion where oscillation or size change of the solution was large, the convergence rate was significantly lower than that in other regions.

In the 1D problem, we have defined the area of the domain to reach a relatively longer region, i.e., we have defined the domain as [0, 2]. In this case of domain region, we found that the ordinary ANN with some small numbers of hidden units failed to converge to the right solution. For this reason, as a special case of the solution to reveal the effectiveness of DDM applied for the solution type made of ANN structure, ANN solution made of some hundreds of hidden units was used for comparison to show the effectiveness of DDM. In the 1D problem, we found that, even for a hardly learnable solution due to some touch conditions regarding the domain, DDM could technically provide guarantees to reach the right convergence.

### 2D problem: effects of basis reconstruction

In this section, numerical results for 2D elliptic PDEs are presented.

As a test problem, we solved the type of Poisson equations given in Eq. (16) with Dirichlet boundary condition for the domain $\Omega ={\left[\text{0,1}\right]}^{2}$. As our test solution, we defined $u=u\left(x\right)$, for $x=({x}_{1},{x}_{2})\in\Omega$, as follows:

18 $$u\left(x\right)=\frac{1}{{e}^{\pi }-{e}^{-\pi }}\text{sin}\left(\pi {x}_{1}\right)\left[{e}^{\pi {x}_{2}}-{e}^{-\pi {x}_{2}}\right].$$

For the structure of the ANN on each subdomain, we set the number of hidden units to be 6, 8, or 10 and applied drop-out^41^ of 1, 3, or 5, respectively. As in the case of the 1D problem, we separated the domain and employed the same amount of hidden units and drop-out on subdomains for each test. The parameter $\upepsilon$ of the BR’s nullifying range was set to be 0.05, 0.1, 0.2, 0.3, and 0.4. Settings of the ANN on each subdomain for the experiments are listed in Table 1. Note that the initial weight parameters of the ANN were given randomly since we intended to verify prominent and consistent effects of methods even in random situations. The ratio of the training epochs at the boundary, subdomain’s interface, and the governing equation was set to be 40:2:2, i.e., the training took place at boundary nodes for 40 epochs, then for 2 epochs at the interface, and then 2 epochs for the governing equation on the interior nodes of each subdomain.

**Table 1:** Settings and relevant parameters of the ANN on each subdomain.

Hidden units	Drop-out	Training data	Learning rate	Epochs of pretraining	Training ratio
6	1	21 × 21	0.0001	10,000	40:2:2 (B:I:G)
8	3	21 × 21			
10	5	21 × 21			

**Figure 8:**
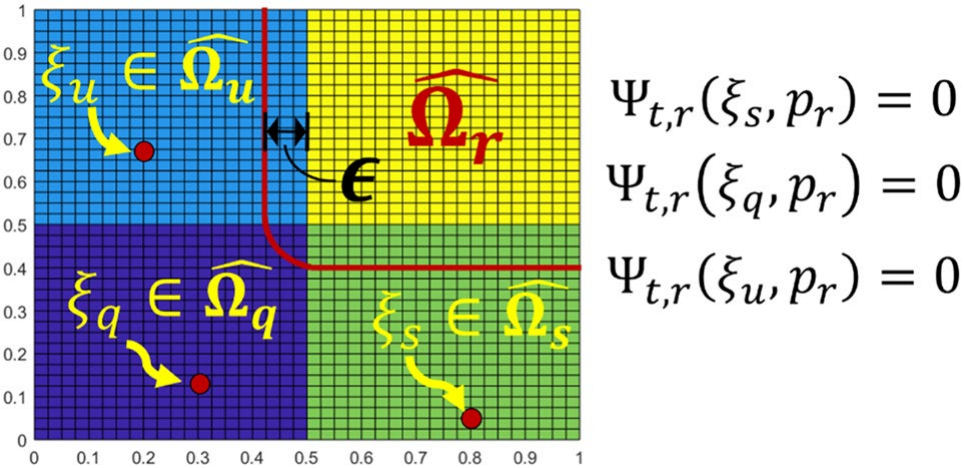
The basis reconstruction scheme with parameter $\upepsilon$ on a $2\times 2$ square subdomain structure.

The basis reconstruction scheme on the $2\times 2$ square subdomains of our test problem is illustrated in Fig. 8.

**Figure 9:**
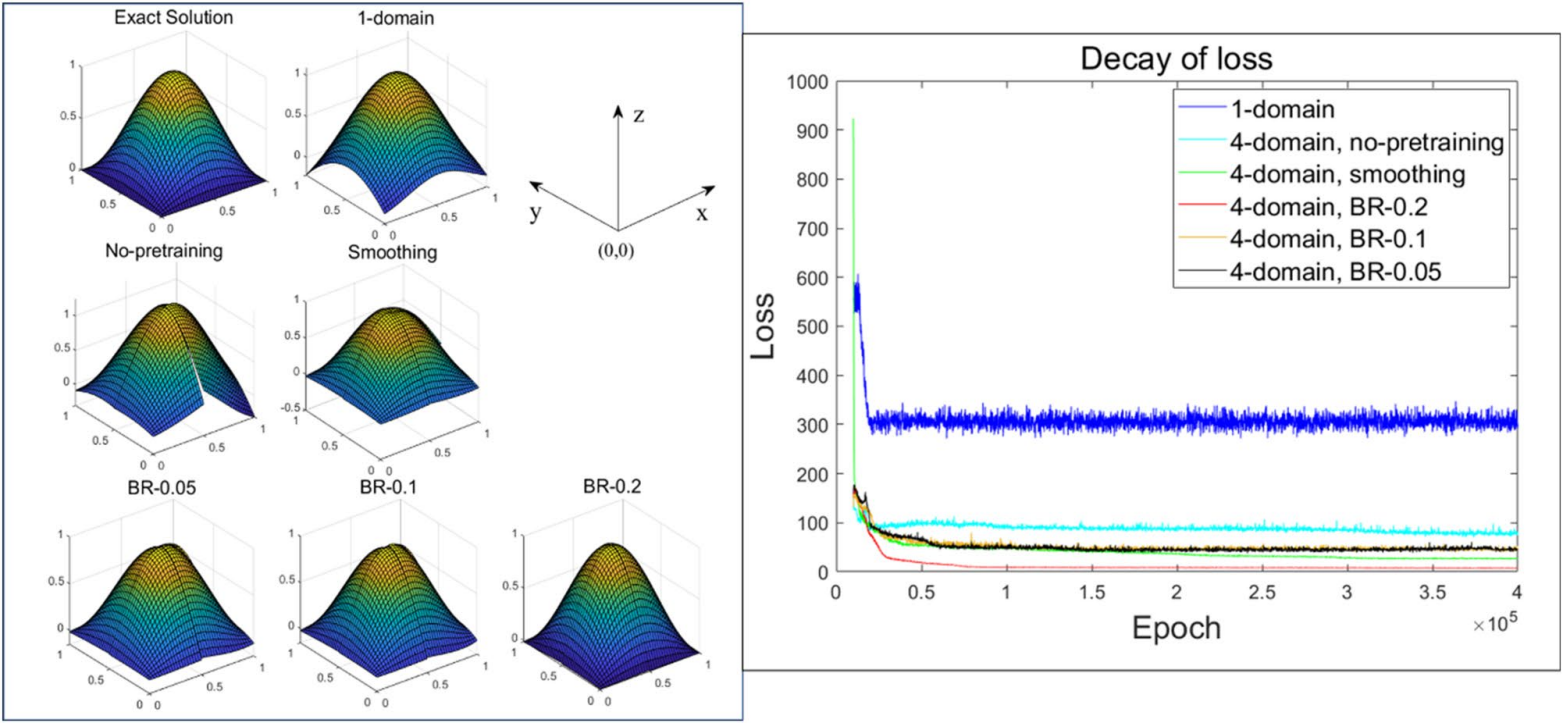
Results of using six hidden units (left) and a comparison of the decay of the loss (right) for both the 1-domain and 4-subdomain methods (no-pretraining, smoothing, BR-0.05, BR-0.1, BR-0.2).

In Fig. 9, the estimated solutions from the case of 6 hidden units are depicted in the subplots (on the left-hand side) with a decaying process of the loss function (on the right-hand side). The solution estimated on the single domain model with 6 hidden units is denoted as ‘1-domain’. The ordinary DDM model without pretraining is ‘No-pretraining’. The pretraining model of DDM with only smoothing is ‘Smoothing’. DDMs of BR methods with $\upepsilon$ of 0.05, 0.1, 0.2 are indicated as ‘BR-0.05’, ‘BR-0.1’, and ‘BR-0.2’, respectively. For the case of ‘1-domain’, the solution estimation was incomplete and the loss remained high, suggesting that shortages of the numerical basis in the ANN caused the estimation to fail. For other methods using DDM structures, the loss declined. These methods relaxed the shortages in the approximation basis of the single domain model. Hence, from a computational effectiveness perspective, when one applies parallel processors, DDM models might improve the convergence rate as independent calculators (processors) are distributed to subdomains, which may save training costs per processor as each processor’s allocated portion of the training data is reduced as the domain is divided (i.e., the overall order of the basis is strengthened whereas the training load on each processor is relieved)^39,40^.

**Figure 10:**
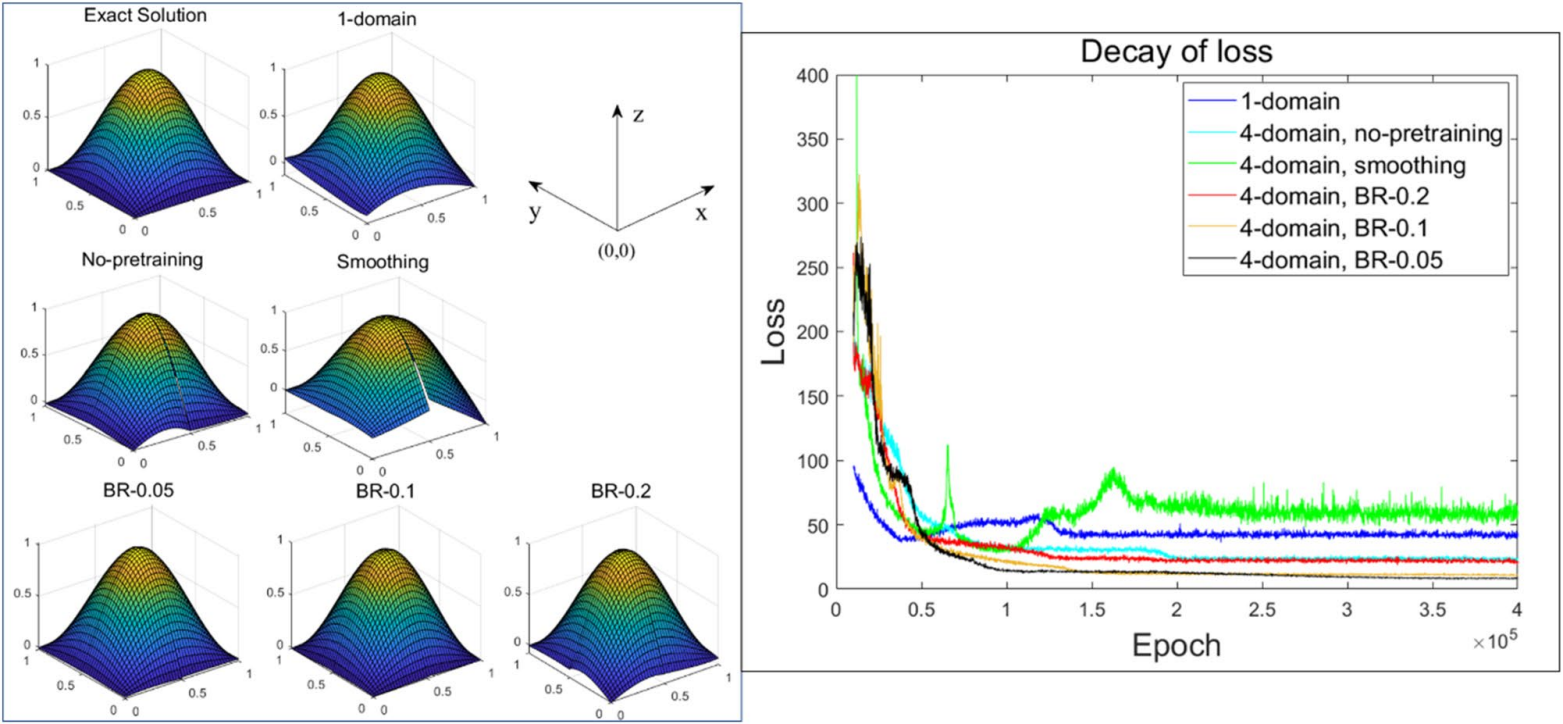
Results for 8 hidden units (left) and comparison of the decay of loss (right) for both the 1-domain and 4-subdomain methods (no-pretraining, smoothing, BR-0.05, BR-0.1, BR-0.2).

**Figure 11:**
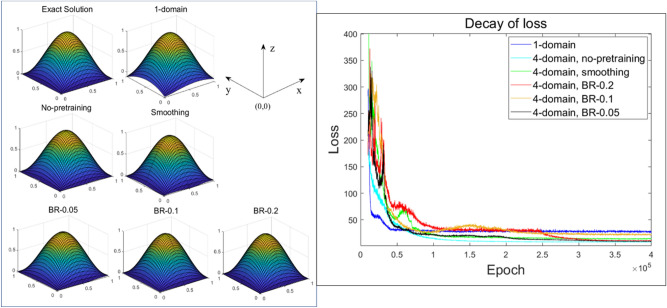
Results for 10 hidden units (left) and comparison of the decay of loss (right) for both the 1-domain and 4-subdomain methods (no-pretraining, smoothing, BR-0.05, BR-0.1, BR-0.2).

To demonstrate further, in Figs. 10 and 11, configurations of estimated solutions and the loss along epochs are given for cases with 8 and 10 hidden units, respectively. It was found that the single domain approximation method still struggled to reduce the loss. DDM methods were comparatively more computationally powerful, although the dominance among them was indiscriminate when the hidden layer’s size was increased.

As shown in Figs. 9, 10 and 11, the DDM-based lower-order ANN model had a more convergent solution than a higher-order single domain model. When training is performed using a given input data, computational costs for both the DDM-based model and the single domain model would be the same if both models’ numbers of hidden nodes are the same. In our work, we focused on the computational efficiency of our proposed DDM technique since single-domain models might have difficulty to obtain the right solutions for some problems while our proposed methods were validated to handle these problems.

**Figure 12:**
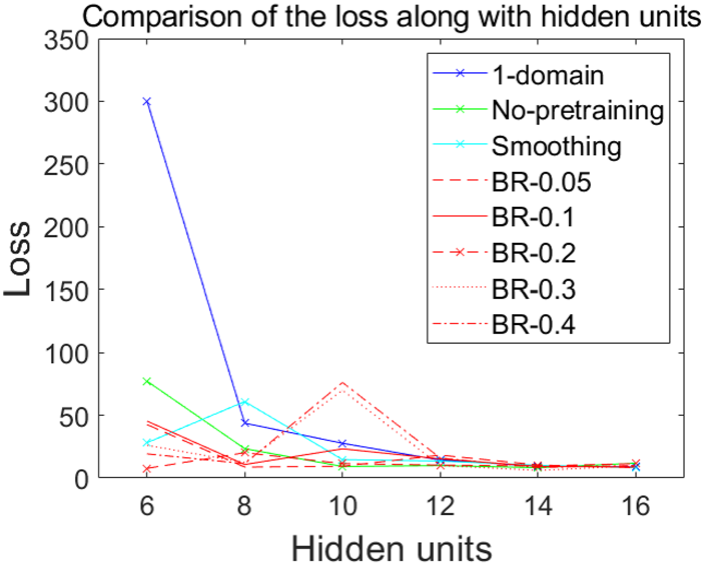
Comparison of the 1-domain method and the 4-subdomain method (no-pretraining, smoothing, BR-0.05, BR-0.1, BR-0.2, BR-0.3, BR-0.4).

Results of comparing different DDM models are presented in Fig. 12, which shows resulting values of the loss at the end of each training with increasing number of hidden units. As shown in Fig. 12, when the size of hidden layers is increased, results are improved. However, the problem is that weight parameters that generate hidden units’ values might be poorly posed with only a few hidden layers. This causes the training process to have shortages of numerical bases. Especially for the BR method with $\upepsilon$ of 0.3 and 0.4, values of the loss were highly unstable. This suggests that the value of $\upepsilon$ may affect the ANN’s ability to estimate accurately. Of course, more reliable evidence of $\upepsilon$’s effect needs to be established by performing relevant well-developed experiments. In the next subsection, we will present that, by pretraining with the BR method, the smoothness of the basis function generated by the hidden layer is improved. Hence, we can interpret that the effect of $\upepsilon$ can cause the BR method to enrich the order of polynomials which shape the basis.

### Comparing polynomial degrees using the ${{\varvec{H}}}_{2}$ semi-norm

We confirmed effects of using the ${H}_{2}$ semi-norm to compare degrees of polynomials in the basis as a measure of smoothness according to pretraining. Although we did not investigate all of the basis-degree with the measure in the ${H}_{2}$ semi-norm reflecting indirect comparison of the strength of the basis, our numerical results gave us confidence that the proposed pretraining method might help the initial basis reform its polynomials to higher degrees.

**Figure 13:**
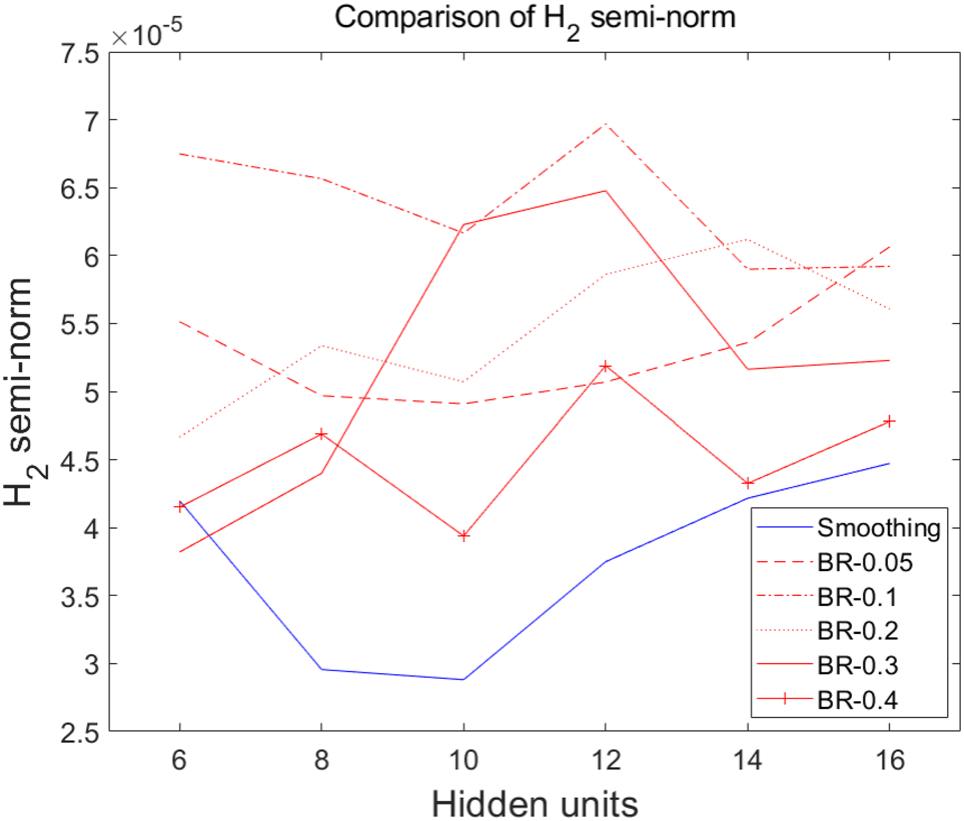
${H}_{2}$ semi-norm comparison for different versions of pretraining models according to the numbers of hidden units in $x$-axis.

**Figure 14:**
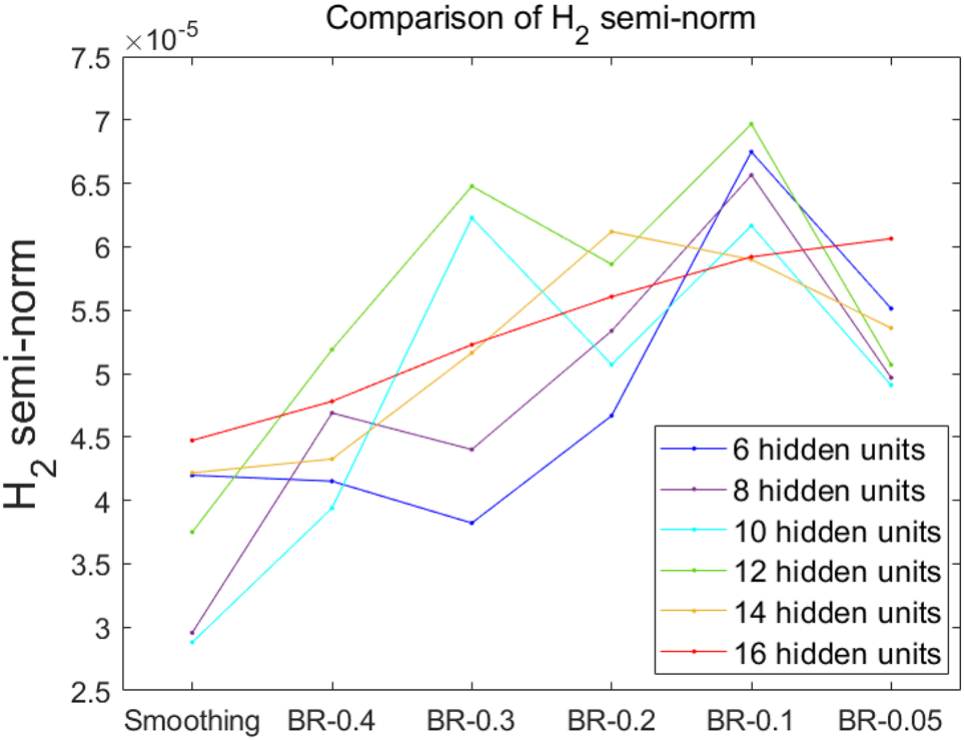
${H}_{2}$ semi-norm comparison for different numbers of hidden units according to different versions of pretraining models in $x$-axis.

From the structure of the ANN, we know that the solution is a linear combination of the outputs of activated hidden units. Hence, the ${H}_{2}$ semi-norm of functions shaped by the activation of the hidden units, i.e., the measure of the degree of the polynomials of each hidden unit’s activation function, can be considered as a measure of the degree of the polynomials of the numerical basis of the ANN. Precisely, we measured the ${H}_{2}$ semi-norm of one hidden unit’s output values activated in each subdomain. The sum of the values of the hidden units on their subdomains was then added to make a total measure (see Table 2). In Table 2, the mean of the ${H}_{2}$ semi-norm is listed as the larger number of hidden units used in the BR method will naturally increase values of the ${H}_{2}$ semi-norm (H) compared to the smoothing method. In Figs. 13 and 14, values listed in Table 2 are plotted. Figure 13 (where the x-axis is H) clearly shows that the values of the ${H}_{2}$ semi-norm for the BR method are almost more than those for the smoothing method. In Fig. 14, the x-axis denotes the method of pretraining. Values tended to increase as $\upepsilon$ decreased. This means that the basis of BR with low values of $\upepsilon$ has improved its polynomial degrees as a result of the pretraining. Results shown in Fig. 12 (where the BR method with $\upepsilon =0.3$ and 0.4 generated a relatively low-quality decay of the loss) could be interpreted that the BR method with a relatively low $\upepsilon$ could give a benefit to fitting the solution’s estimation in higher orders due to enrichment of the basis.

**Table 2:** ${H}_{2}$ semi-norm comparison along with the number of hidden units for several pretraining models.

	H					
	6	8	10	12	14	16
Smoothing	4.198e−05	2.956e−05	2.881e−05	3.750e−05	4.217e−05	4.473e−05
BR0.05	5.513e−05	4.968e−05	4.908e−05	5.069e−05	5.359e−05	6.064e−05
BR0.1	6.748e−05	6.565e−05	6.165e−05	6.968e−05	5.899e−05	5.920e−05
BR0.2	4.667e−05	5.336e−05	5.071e−05	5.862e−05	6.119e−05	5.606e−05
BR0.3	3.821e−05	4.401e−05	6.228e−05	6.477e−05	5.163e−05	5.228e−05
BR0.4	4.151e−05	4.689e−05	3.939e−05	5.189e−05	4.326e−05	4.781e−05

Note that the computation of the ${H}_{2}$ semi-norm on the domain is conducted as follows.

Let us define the output of the activation of the hidden units as ${{\hbar }}_{k}$ such that

19 $${{\hbar }}_{k}=\sigma \left({w}_{k}\overline{x }\right),$$

where ${w}_{k}\in {\mathbb{R}}^{H\times \left(d+1\right)}$ denotes learnable weights defined in Eq. (1) and the subscript $k$ denotes the index of subdomains. The discrete form of the second-order derivative is straightforward as shown in Eq. (20):

20 $$\frac{{\partial }^{2}{{\hbar }}_{k}(\xi )}{\partial x{\left(i\right)}^{2}}=\frac{{{\hbar }}_{k}\left({\xi }^{i,+\delta }\right)-2{{\hbar }}_{k}\left(\xi \right)+{{\hbar }}_{k}\left({\xi }^{i,-\delta }\right)}{{\delta }^{2}} ,$$

where, for a positive real number $\delta >0$, ${\xi }_{i,-\delta }$ and ${\xi }_{i,+\delta }$ are defined as varying the $i$th component of $\xi$ by $\pm \delta$, i.e., ${\xi }^{i,-\delta }=(\xi (1),\xi (2),\dots ,\xi (i)-\delta ,\dots ,\xi (d))$, ${\xi }^{i,+\delta }=(\xi \left(1\right),\xi \left(2\right),\cdots ,\xi \left(i\right)+\delta ,\dots ,\xi (d))$.

The measure of the ${H}_{2}$ semi-norm of ${{\hbar }}_{k}$, denoted ${\left|{{\hbar }}_{k}\right|}_{2}$, is defined by the following:

21 $${\left|{{\hbar }}_{k}\right|}_{2}=\sum \limits_{j=1}^{H}{\left\{\delta \sum \limits_{x\in {\Omega }_{k}}{\left(\sum \limits_{i=1}^{d}\frac{{\partial }^{2}{{\hbar }}_{i}}{\partial x{\left(i\right)}^{2}}\right)}^{2}\right\}}^{1/2 }.$$

We summed all individual ${\left|{{\hbar }}_{k}\right|}_{2}$ so that we could have ${\sum }_{k}{\left|{{\hbar }}_{k}\right|}_{2}$ for values listed in Table 2.

### Verification of the learning efficiency for general ANN tasks by simulation

**Figure 15:**
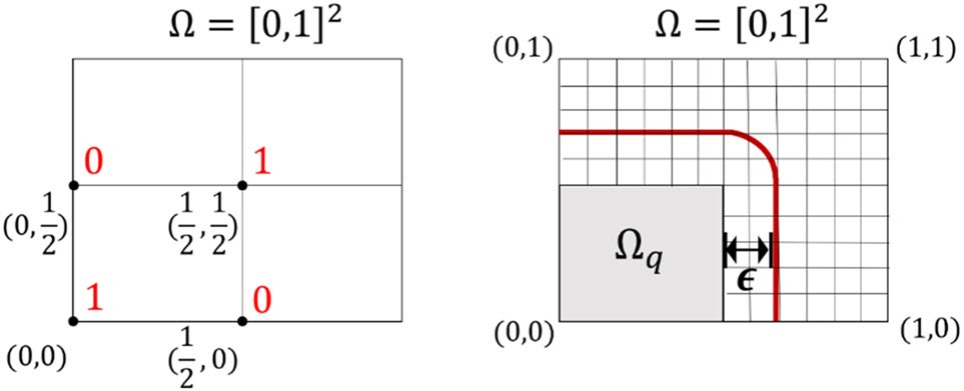
The domain of the XOR problem, data points with labels in red letters (left), and the domain structure to apply the BR method with $\upepsilon$ (right).

If the BR method can be effectively applied to the estimation of solutions of PDEs, it stands to reason that it may also be effectively applied to a general task of machine learning with ANNs. To generally prove that the BR method can enhance the performance of ANNs as a tool for machine learning, we tested the BR method on the problem of the differentiation of the exclusive OR (XOR) logic gate, a well-known low-dimensional problem of machine learning on which to apply an ANN. To arrange the problem of XOR in a way that could fit the concept of the BR method, we reorganized the dataset in the computational domain $\Omega \subset {\mathbb{R}}^{H}$, where $\Omega ={\left[0, 1\right]}^{2}$, as follows (see Fig. 15).

The XOR dataset is organized as (0,0), (1/2, 0), (0, 1/2), (1/2, 1/2), with labels of 1, 0, 0, 1, respectively. We defined the domain ${\Omega }_{q}$ as ${\Omega }_{q}={\left[0, 1/2\right]}^{2}$ which included the dataset (see Fig. 15) and denoted $\widehat{{\Omega }_{q}}$ as  $\widehat{{\Omega }_{q}}$ = [(0,0), (1/2, 0), (0, 1/2), (1/2, 1/2)] with the set of labels Y = [1, 0, 0, 1]. Note that the definition of $\widehat{{\Omega }_{q}^{\upepsilon }}$ is given in Eq. (15).

**Figure 16:**
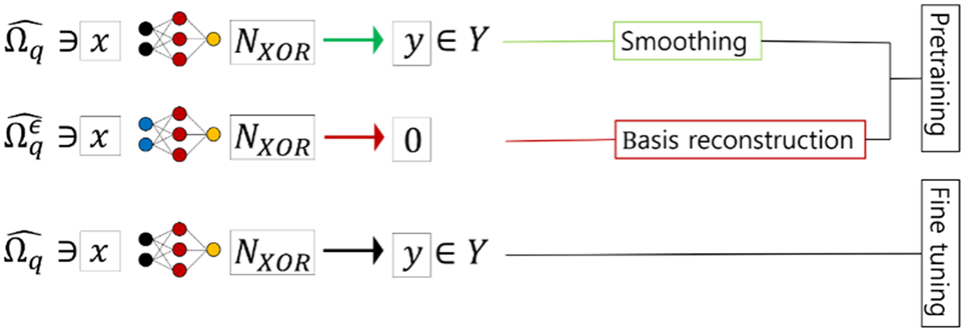
The BR method applied to the XOR problem.

Now, we can employ the BR scheme which is illustrated in Fig. 16. Since BR is a pretraining scheme, we determined the efficiency as a function of the number of epochs in the pretraining process. In our experiments, we set the number of pretraining epochs to be 500, 1000, 2000, and 3000. Results are compared with the ordinary training scheme which does not apply any pretraining method. We discretized each subdomain of $\Omega$ except $\widehat{{\Omega }_{q}}$ into 21 equi-spatial nodes along each axis so that there were about 400 nodes in total.

**Figure 17:**
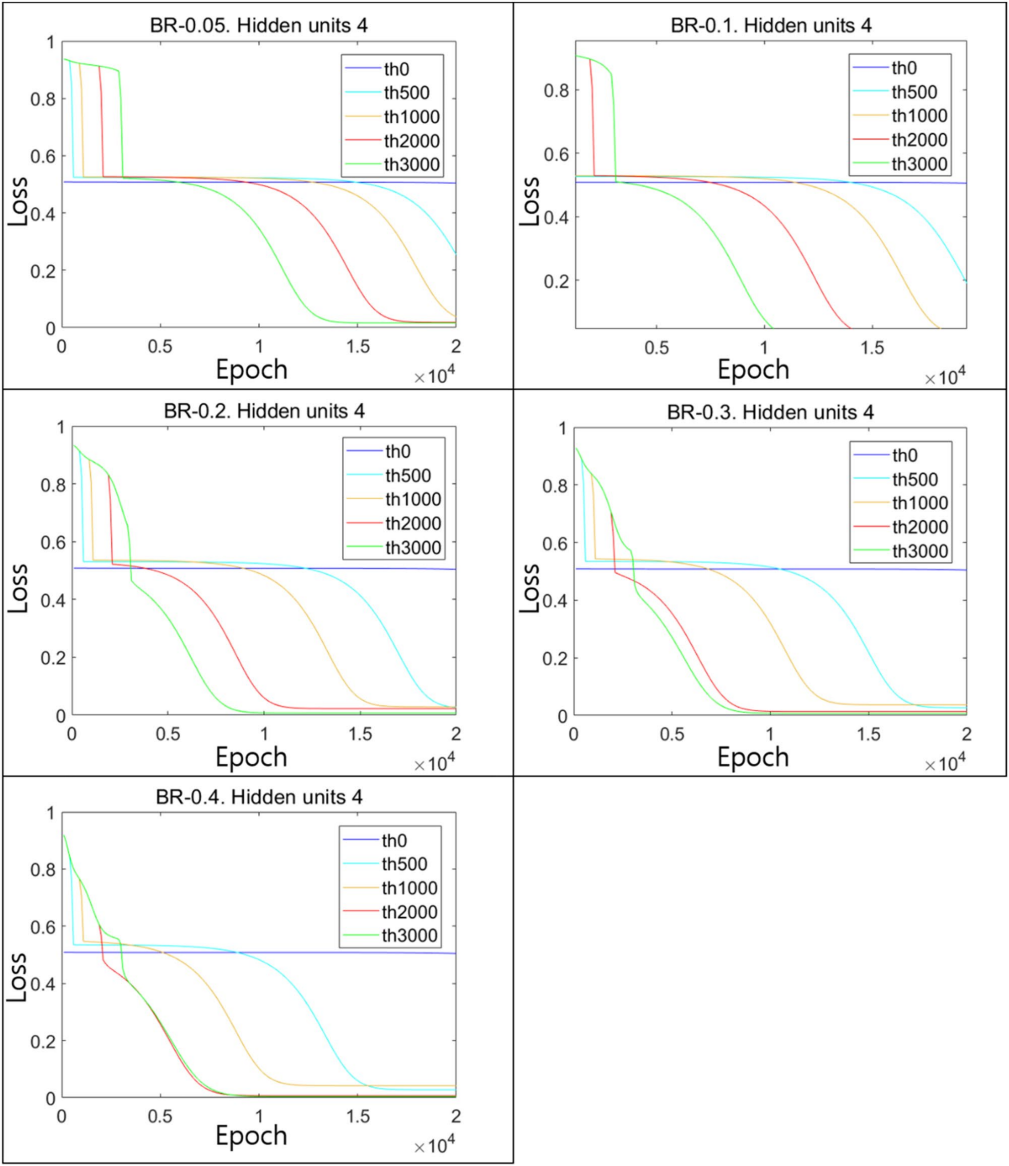
Results for the BR method with various sizes of $\upepsilon$ compared to the ordinary training scheme for the case with 4-hidden units.

In Fig. 17, results of the experiments for BR with varying $\upepsilon$ are shown for the case with 4 hidden units. The values of $\upepsilon$ used were 0.05, 0.1, 0.2, 0.3, and 0.4. In the plots, data for 500, 1000, 2000, and 3000 epochs of pretraining are indicated as PT500, PT1000, PT2000, and PT3000, respectively. The ordinary training scheme which had no pretraining process was denoted as PT0. Note that initial values of neural weights were set to be the same for each case since we aimed to verify the progression as we increased epochs of the pretraining of each method. Our results revealed that as the number of epochs of pretraining increased, the convergence of the loss to 0 was accelerated. As the size of $\upepsilon$ increased, the speed of convergence was further accelerated. However, for the case of PT0, the convergence progress appeared to stop in the middle and the training failed to optimize the loss.

**Figure 18:**
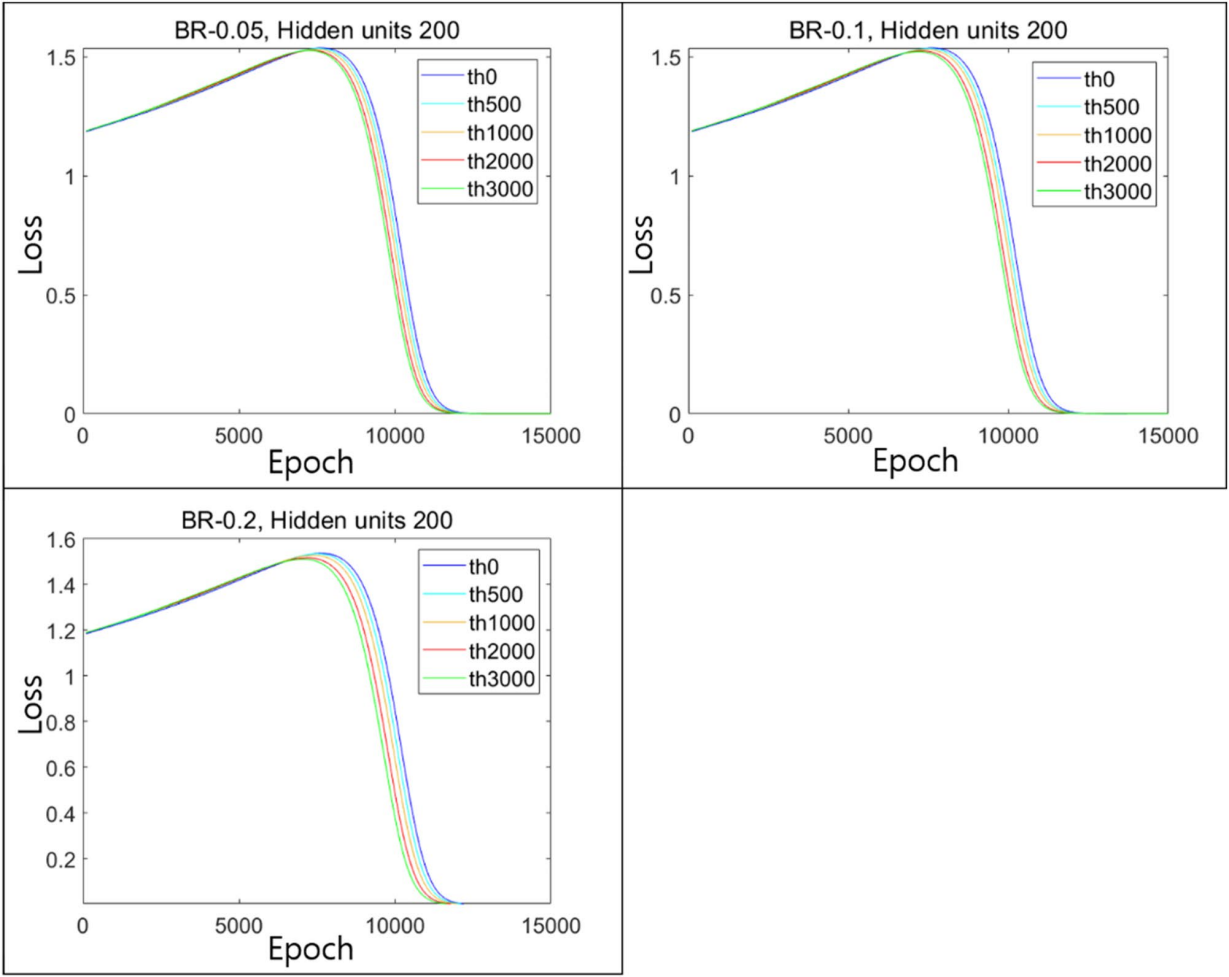
Results of the BR method with various sizes of $\upepsilon$ for the case of 200-hidden units.

To make a comparison in the case of a larger hidden layer, we repeated the experiments with 200 hidden units and plotted the results for ϵ = 0.05, 0.1, and 0.2 as shown in Fig. 18. Similar to previous results with 4-hidden units, as the number of pretraining epochs increased, the convergence of the loss accelerated, although the difference in the speed of convergence was insignificant (Fig. 18). Experimental results shown above suggest that the BR technique applied in the pretraining seems to help the ANN model estimate the solution to the XOR problem. Furthermore, the finding that the convergence speed increased with the number of epochs in the pretraining showed us that the training results might not be caused by a random phenomenon with some vague factors and that the BR method strongly contributed to the effective utilization of the optimization power of the hidden units.

**Figure 19:**
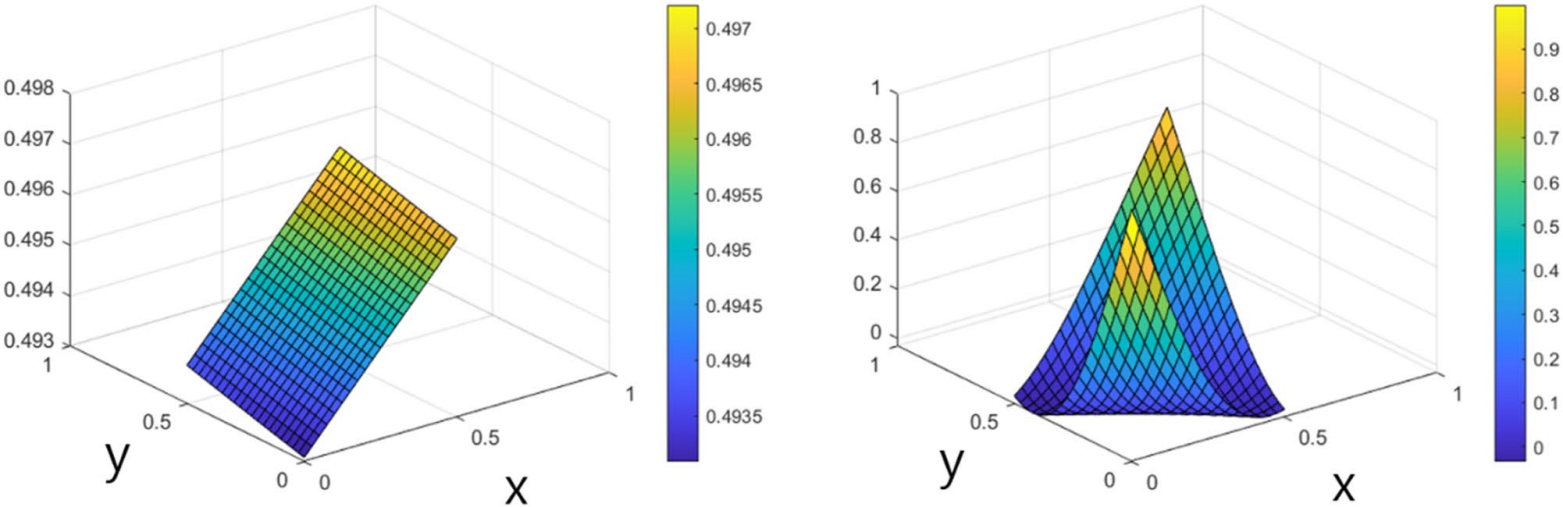
Resulting approximations with 4-hidden units made by methods of PT0 (left) and PT3000 (right).

On the other hand, to demonstrate that the basis reconstruction method improved the approximation power of ANNs, the basis function of each hidden unit was developed into a higher state of the degree of polynomials so that the estimation power of the hidden layer could be improved. The approximated function representing the output function of the XOR logic gate given in Fig. 19 shows a wrong approximation of XOR by PT0 on the left and a higher-quality approximation on the right. The solution of the XOR function might have a high order of curvature’s smoothness. Thus, the approximating numerical basis functions of the ANN also need to be in the form of some high-degree polynomials. With prerequisite knowledge, we observed shapes of the hidden units’ pretrained activation. Since the output model of the ANN is given by the linear combination of the hidden units’ activation, a comparison of the hidden units’ activations may give us some fine insight to understand properties of the ANN.

## Conclusion

The estimation's accuracy using NN depends on the solution’s analytic complexity. If the solution consists of high order polynomials, it needs to employ high order hidden units in NN because the number of hidden units determines the estimation’s order of polynomials. In this study as basic research, we applied the proposed method for a single hidden layered architecture based on the universal approximation theory of the single hidden layered NN^25^. In our study of the implementation of an ANN-PDE solver, we proposed a pretraining version of ANN-based DDM which used a basis reconstruction technique where weight parameters in the hidden layer were rearranged to enrich the quality of the numerical basis and estimated solutions. The BR method was implemented by setting the output of the ANN to be zero on the neighboring subdomains. The main idea is that, as the ANN is nullified on other subdomains, eventually the core degrees of polynomials constituting the hidden units’ numerical basis might be rearranged to center on their main subdomains. Experiments were conducted with one- and two-dimensional Poisson’s equations for the XOR problem. When the BR method was applied to PDE problems, it was observed that the BR method enhanced the computing power to accelerate the rate at which poor estimations of an ANN on single domains were improved in the DDM model by reducing the computational costs relative to single-domain models. On the other hand, to numerically demonstrate the basis reconstruction property and benefits for the weight parameter’s initialization, we compared the after-pretraining status of the hidden units’ activation in the XOR problem. Results showed that degrees of polynomials of activations by the BR method, understood by measuring the curvature, were enriched. This gives a superior approximation compared with ordinary training procedures.

In this work of developing DDM in the structure of ANNs, the basis reconstructing treatment was effective for estimating solutions to PDEs. It could also be applied well to general machine learning tasks. Thus, it may be applicable as a tool for subjects such as regularization, weight initialization, and speeding computations in training.

Nowadays, lots of research have been conducted applying more deeper architectures. For instance, researchers have used ResNet^42^ which provides more promising performances than any basic deep neural network (DNN)-based architecture due to the skip-connection’s property to find optimal number of hidden layers without too laborious tests made for some normal DNN architectures. However, for a deeper architecture of neural network, we have to apply the proposed method and test it to know how effective it will be in future works.
