# Publisher Correction: A pretraining domain decomposition method using artificial neural networks to solve elliptic PDE boundary value problems

**DOI:** 10.1038/s41598-022-21950-6

**Published:** 2022-10-14

**Authors:** Jeong-Kweon Seo

**Affiliations:** Institute of Data Science, Korea University, 145 Anam-ro, Seongbuk-gu, Seoul, 02841 South Korea

Correction to: *Scientific Reports* 10.1038/s41598-022-18315-4, published online 17 August 2022

The original online version of this Article contained an error in the Reference List, where the order of the references was incorrect.

The original Article has been corrected.

